# Large Language Models for Synthetic Dataset Generation of Cybersecurity Indicators of Compromise

**DOI:** 10.3390/s25092825

**Published:** 2025-04-30

**Authors:** Ashwaq Almorjan, Mohammed Basheri, Miada Almasre

**Affiliations:** Department of Information Technology, Faculty of Computing and Information Technology, King Abdulaziz University, Jeddah 21589, Saudi Arabia

**Keywords:** large language model, cybersecurity, indicators of compromise, domain knowledge, social media, synthetic data generation, cyber threat intelligence, ChatGPT

## Abstract

In the field of Cyber Threat Intelligence (CTI), the scarcity of high-quality and labelled datasets that include Indicators of Compromise (IoCs) impact the design and implementation of robust predictive models that are capable of classifying IoCs in online communication, specifically in social media contexts where users are potentially highly exposed to cyber threats. Thus, the generation of high-quality synthetic datasets can be utilized to fill this gap and develop effective CTI systems. Therefore, this study aims to fine-tune OpenAI’s Large Language Model (LLM), Gpt-3.5, to generate a synthetic dataset that replicates the style of a real social media curated dataset, as well as incorporates select IoCs as domain knowledge. Four machine-learning (ML) and deep-learning (DL) models were evaluated on two generated datasets (one with 4000 instances and the other with 12,000). The results indicated that, on the 4000-instance dataset, the Dense Neural Network (DenseNN) outputs the highest accuracy (77%), while on the 12,000-instance dataset, Logistic Regression (LR) achieved the highest accuracy of 82%. This study highlights the potential of integrating fine-tuned LLMs with domain-specific knowledge to create high-quality synthetic data. The main contribution of this research is in the adoption of fine-tuning of an LLM, Gpt-3.5, using real social media datasets and curated IoC domain knowledge, which is expected to improve the process of synthetic dataset generation and later IoC extraction and classification, offering a realistic and novel resource for cybersecurity applications.

## 1. Introduction

Cybersecurity becomes an essential concern among all sectors as digital systems continue to grow in complexity and scale. Alongside the increasing connectivity of services such as IoT-based monitoring systems [1], devices, IT Infrastructure [2] and applications, the scope of attacks and incidents has notably expanded. These threats not only disturb service accessibility but also compromise sensitive data, raise financial costs, and damage enterprise reputation. Recently, social media platforms have appeared as fruitful grounds for distributing malware, obtaining personal information, and arranging global cyber campaigns. The nature of unstructured content on these platforms creates distinctive challenges for incident response and threat detection.

The continuous growth of cyber threats requires novel approaches to detect them since traditional methods of identifying and managing these threats, such as rule or signature-based security systems, have become increasingly ineffective against fast-developing attack tactics. Cyber Threat Intelligence (CTI) has become a valuable method in the hands of cybersecurity experts as it provides insights about both existing and emerging threats and enables the adoption of proactive defensive techniques. CTI not only aids in threat detection but also in strategic analysis of threat actors and their tactics. Hence, it greatly enhances organizational resilience to cyber attacks [3,4]. In this scenario, Indicators of Compromise (IoC) are considered tangible evidence, which indicates a potential/ongoing cyber threat. These IoCs play a significant role in the detection, analysis, and response to malicious activities inside a network or system [5].

Despite its potential, one of the most significant challenges in CTI is the lack of topical high-quality labelled datasets for training predictive models, especially ones representing human interactions on social media platforms, for example. Such cyber methods of interactions are widely used on both the personal and organizational levels, which leads to the greatest impact in case cyber security threats are being transmitted through them [5,6].

Most significantly, labelled datasets for IoCs, which are basically traces that indicate a specific security breach, such as IP addresses, hashes, domain name, emails, URLs, and keywords, are also scarce [5] and require effort to annotate [7]. This constitutes a limitation to the development of effective machine-learning (ML) and deep-learning (DL) models that can detect or extract IoCs.

Generating synthetic data, research indicates, is a feasible solution to this challenge, permitting the simulation of realistic data to expand the limited real-world data available [8]. Previous studies demonstrated that synthetic data could enhance the performance of cyber threat intelligence, particularly in generating malicious activities within digital text formats [9,10,11]. We assume that large language models (LLMs) being trained on large original datasets have learned the persistent styles of most human-interaction types. This peculiar attribute of LLMs is useful if one wants to develop natural, unstructured textual datasets focusing on specific topics or styles (e.g., social media comments), for example. This entails, as research in the domain of LLM data generation described, fine-tuning an LLM (like ChatGPT) on an original dataset that is labeled (by social platform, for instance) to drive the LLM to identify and emulate the style of each comment based on the platform. In addition, these LLMs can learn from domain knowledge, such as a database of IoCs, and use it to drive the generation of synthetic datasets [8].

The research makes contributions in the domain of fine-tuning an LLM using two primary sources: real social media datasets and curated IoC domain knowledge. The objective is to enhance the generation of synthetic datasets and the subsequent extraction and classification of IoCs. Our contributions can be outlined in the following:

1. By fine-tuning the LLM on the curated social media datasets, our model becomes adept at identifying the distinct patterns, linguistic features, and communication styles present in such discourse. We argue this includes the detection of malicious behaviour, activities, or content within user interactions, which could otherwise go unnoticed by traditional threat detection models and systems.
2. The generation of a synthetic dataset that emulates the style of social media content and IoC knowledge, which can be used to simulate a wide range of attack scenarios and behaviours. This dataset can be used to test cybersecurity systems and methodologies in a controlled, privacy-conscious manner while maintaining high relevance to real-world situations.
3. When trained on this synthetic data, ML and DL models can learn to identify and classify IoCs more effectively, improving their performance across different types of attack vectors and threat patterns. This approach enhances the generalization and adaptability of these models, enabling them to detect previously unseen or emerging threats with greater accuracy and speed.

The assumption is that with the generation of synthetic datasets that incorporate IoCs, the detection of these indicators can be improved, hence the design of advanced CTI systems. Thus, the aim of this research is two-fold: (1) fine-tuning an LLM to generate a synthetic dataset that emulates the style of curated real social media datasets, incorporates IoC domain knowledge, and labels it for both platform and type of IoC, (2) evaluating selected ML and DL models on the generated dataset to detect IoCs. This can further be broken into the following objectives:

1. Curate public social media comments datasets, clean and pre-process them, then label the comments in each dataset using the platform name,
2. Fine-tune and evaluate ChatGPT 3.5 on the dataset to classify the comments by platform,
3. Use a curated IoC domain knowledge and the previously fine-tuned GPT model to generate a synthetic dataset labelled by both social media platform name and IoC type,
4. Evaluate the performance of selected ML and DL models on the generated dataset to classify comments using IoC types.

The proposed solution can provide researchers and the industry with a method for overcoming the issue of a lack of IoC datasets, which is related to specific platforms like social media. It can be part of enhanced threat hunting systems that work cross-platform to detect IoCs using contextually relevant attributes.

Henceforward, the paper in Section 2 discusses related work on IoC detection and synthetic dataset generation was reviewed. Section 3 presented the implementation and results, which involves dataset curation and development, fine-tuning the ChatGPT 3.5 LLM, synthetic data generation using IoC domain knowledge, and an evaluation of selected ML and DL models on the generated dataset. Finally, the study concluded by summarizing the findings and suggesting directions for future research.

## 2. Related Works

This section reviews major areas of related work that inform the methodology of this research on cybersecurity threat detection, IoC detection, and synthetic dataset generation.

### 2.1. Cybersecurity Threat Detection

Cybersecurity threats are rapidly growing in different technology fields, requiring targeted research and explanations. Recent studies have investigated risks in Digital Twins, IoT systems, workstation environments, cloud computing [12], and wireless sensor networks, additionally exploring the role of ML in threat detection and prevention. These studies present important insights into existing vulnerabilities and defense approaches.

For example, Otoom [13] classifies cybersecurity risks in Digital Twin (DT) environments within Cyber–Physical Systems (CPSs) and suggests risk management strategies to improve the security of DTs. The results showed that data reliability was the most generic concern with DDoS attacks found in 38% of tasks, and 56% of the literature was on authentication weaknesses. Also, the findings of the study indicated that blockchain (21%) and ML-based anomaly detection (19%) are emerging solutions that can be utilized to enhance risk-auditing strategies to DT implementations in fields like healthcare, smart cities, manufacturing, and IoT [13].

Similarly, Almuqren [14] discusses cybersecurity challenges in the domain of the Internet of Things (IoT) architecture to classify threats, assess mitigation approaches, and point out future directions. It categorizes threats of the IoT application and network layers. In the application layer, the result showed that 30% of threats, such as malware, spyware, and virus attacks, were reported; afterwards, 20% of malicious code, 15% of phishing, and 10% of botnets and cross-site scripting were reported. However, in the network layer, 36% of denial-of-service (DoS) and replay attacks each were represented incidents, as well as 27% of man-in-the-middle attacks and 9% each of Sybil and selective forwarding. Thus, this paper highlighted the value of developing a strong defense for each layer [14].

Mousa and Shehab [15] classify threats, vulnerabilities, and countermeasures in workstation situations to prove the selection of useful cybersecurity controls. Utilizing a content analysis approach, the study follows four main phases: finding components, threats, vulnerabilities, and countermeasures. The analysis showed that man-in-the-middle attacks and malware are the most general threats, accounting for 25% and 27% of incidents, respectively. Based on vulnerabilities, weak access control (20%) and unpatched software (21%) are the most common. The study highlights encryption, access controls, and firewalls as critical mitigation strategies. The results proposed a robust foundation to improve cybersecurity policies and risk mitigation approaches.

Alshuaibi et al. [16] studied the function of ML methods to find key cybersecurity issues. The authors’ purpose is to recognize effective ML approaches for mitigating threats. The study classified ML algorithms into supervised, unsupervised, and reinforcement learning models and represented them to specific cyber threats. Particularly, supervised algorithms like (SVM) and Decision Trees are revealed to be mainly effective in intrusion detection, while unsupervised models are best in anomaly detection. The paper offered appreciated visions into how ML can improve cyber defense approaches and highlights the necessity for continuous research to adjust to the dynamic cybersecurity environment.

This study by Alotaibi et al. [17] focused on cybersecurity vulnerabilities in Wireless Sensor Networks (WSNs); specifically, on threats such as DoS, Sybil, wormhole, and sinkhole attacks. The main goal is to assess the success of current defense methods and to show the limitations caused by the forced nature of WSNs, containing limited processing power, memory, and energy resources. The authors highlight how essential it is for lightweight and energy-efficient security solutions to be adjustable to the exact architecture of WSNs. The variety of WSN uses creates challenges in developing standardized, successful security protocols.

To summarize, these studies collectively presented an overview of cybersecurity threats among varied environments, each delivering a different domain, challenge, and strategy. All studies highlight the requirement of context-specific solutions and underscore the rising integration of systematic risk assessment and AI in existing cybersecurity research. Table 1 summarizes studies based on the cybersecurity threat detection domain, objectives, models used, and the results.

### 2.2. IoC Detection

In the field of cybersecurity, IoCs are an essential part of threat information for cybersecurity detection attempts. Recent research has investigated various approaches to improve the detection and identification of IoCs using different techniques such as ML or DL. For instance, Zhou et al. [18] applied a neural-based sequence labeling technique that combined bidirectional Long Short-Term Memory (LSTM) and a Conditional Random Field (CRF) layer to detect IoCs from English cybersecurity reports. The study determined high precision, with an average rate of 90.4%. Similarly, another study by Long et al. [19] utilized a multi-head self-attention to increase performance in collecting relevant contextual data from unstructured text, reaching 93.1% on an evaluation English dataset and roughly 83% on a Chinese dataset. Both studies used datasets derived from reports and implemented neural network models, achieving good results in testing their data. However, our study employs various social media data sources to increase the range of detectable IoCs. This approach represents varied and dynamic cyber threat scenarios, essentially improving the robustness and applicability of IoC detection in different circumstances.

Investigating Twitter as a source for CTI, Arikkat et al. [20] studied how IoCs, such as IP addresses, URLs, file hashes, domain addresses, and CVE IDs, can be obtained from Twitter to help in proactive security actions. By applying a Convolutional Neural Network (CNN), tweets were classified to identify valuable threat indicators using different performance indicators like correctness, timeliness, and overlap. In addition, the speed at which Twitter shares IoCs was investigated and compared to traditional threat intelligence services. Moreover, the study classified the Twitter accounts as automated or human-operated to understand the role of bot accounts in publishing cyber threat information. To obtain cyberthreat intelligence from Twitter, there is a study that utilized a multi-task learning model integrating CNN for classifying tweets and a Bidirectional Long Short-Term Memory (BiLSTM) network and Named Entity Recognition (NER) to enhance the effectiveness of threat detection. The outcomes established that the method offered timely and reliable alerts [21]. Through using a combination of graph theory, ML, and text mining to reorganize the process of detecting relevant cyber threat information, Niakanlahiji et al. [22] displayed the “IoCMiner” framework for Twitter to automatically extract IoCs. This is realized by employing a pattern CTI classifier and a set of regular expression rules. The outline showed effective in detecting over 1200 IoCs, containing many malicious URLs that were not listed in the open blacklist dataset. The results of these investigations indicated that Twitter is an efficient platform for gathering related malware IoCs and offered insights into how Twitter can be employed to increase threat intelligence and possibly support in the early detection and prevention of threats.

For the dark-web platform, Murty et al. [23] applied Support-Vector Machines (SVM) for classifying text-based keywords from over 1800 dark-web sites. The outline effectively predicted the sort of content hosted by URLs in this platform. The dataset contains keywords from 1800 manually labeled dark web URLs, delivers robust testing, and reaches precision, recall, and F-measure values of 0.83, 0.90, and 0.96, respectively, on the dataset. Another framework aimed to extract security vulnerabilities and cyber attacks from web text [24]. It utilized a knowledge base called Wikitology, derived from Wikipedia, to extract specific concepts related to vulnerabilities and attacks. This approach is not just for structuring existing descriptions of vulnerabilities but also for detecting new ones by evaluating text streams from diverse sources such as social media or chat rooms. The effectiveness of this approach was tested against the National Vulnerability Database, providing potential in identifying new threats and gathering data on existing issues.

Selecting a testbed dataset as a target, the study by Atluri et al. [25] planned to identify IoCs among five different simulated cyber attacks by applying nine diverse ML models. The dataset comprised 120,025 network packets, classified into benign operations and several types of simulated attacks, such as Man-in-the-Middle and DDoS attacks. The Bagging Decision Trees (BDT) performed with the highest accuracy 94.24%, precision 95%, recall 93%, and F1 score 94%. Similarly, Li et al. [26] introduced a method that combines Latent Semantic Indexing (LSI) for topic indexing, semantic similarity calculations using the ATT&CK taxonomy, and multi-label classification techniques to address the challenge of extracting relevant threat actions from the vast amounts of unstructured text. The method aims to increase the defense capabilities against cyber threats by providing a structured analysis of threat actions reported on different platforms. The method compiled and utilized a labeled dataset related to APT groups, indicating the capability to achieve a precision of 59.50%, a recall ratio of 69.86%, and an F-1 measure of 56.96%. Alternatively, Preuveneers et al. [27] proposed a method to CTI by sharing ML models as IoCs. Unlike traditional IoCs, which usually contain simple labelled attributes like IP addresses, the shared ML models delivered a method for detecting threats, increasing detection capabilities against adversaries’ evasion tactics. The results proved the usefulness of the framework, with the BDT model achieving the highest performance among nine examined ML models.

These studies emphasize the different approaches being investigated in the domain of cybersecurity using ML and other AI models. Each method targets the improvement of threat intelligence and response processes, which are essential in the protection of information in a complex digital world. Table 2 summarizes IoCs detection research based on the model applied, features, IoCs types, data sources, and results.

### 2.3. Synthetic Dataset Generation

Synthetic dataset generation in the cybersecurity domain has obtained significant attention in recent years. Research has determined how synthetic dataset generation can address several cybersecurity challenges. Das et al. [9], for instance, proposed a framework for generating synthetic malicious emails using Natural Language Generation (NLG) methods. This deep-learning technique generates fake emails with malicious content using Recurrent Neural Networks (RNNs). The framework performance was evaluated applying three models: SVM, Naive Bayes (NB), and Logistic Regression (LR). Among these, LR reached the highest accuracy of 91%.

Zacharis et al. [10] established AiCEF, a framework that utilizes named entity recognition (NER) to generate content for cyber exercises. The research applied a large dataset of public cybersecurity articles to predict upcoming threats and create new exercise scenarios. Methods such as NER and topic extraction are utilized to manage information based on a novel ontology called the Cyber Exercise Scenario Ontology (CESO). CESO was employed to rapidly assess both fragments and the last scenario content in the AI-assisted Cyber Exercise Framework. The approach was evaluated by experts among a collection of generated scenarios used in a real exercise. To increase accuracy, several iterations were performed by increasing the annotation dataset and retraining the model. This iterative process was repeated until reaching an F1 score of about 80%.

Adversarial text-generation strategies were investigated by Stilinski et al. [28] for generating synthetic cyber threats to evaluate NLP-based anomaly-detection schemes. The method involves gradient-based approaches, generative models, and processes, generating subtle text variations to fool detection systems. The research detected the benefits and limitations of these methodologies and assessed the model’s performance employing a variety of criteria.

Recent experiments have been done using LLMs for generating a synthetic dataset. For instance, Khosh Kholgh et al. [29] proposed a PAC-GPT framework that utilizes OpenAI’s GPT-3 for generating synthetic network traffic. The PAC-GPT covers two basic modules: a flow generator, which has network packets that capture and regenerate patterns; and a packet generator, which generates a single network packet based on the network flow. It also used a packet generator by LLM and evaluated its performance by applying metrics like accuracy, loss, and success rate. The outcome determined that transformers are successful for synthetic packet generation with minimal fine-tuning. Moreover, a basic command-line interface tool was developed to access the new data-generation strategy for specialists across various areas.

LLMs were applied by Houston [30], using GPT and BERT models to enhance text classification in cybersecurity. It proposes to increase the performance of ML models in classifying textual cybersecurity by employing GPT-2 for generating a synthetic dataset and BERT for semantic encoding. This is intended to address the concerns of a lack of data and increase the accuracy of classification tasks in the domain of cybersecurity. The result of the model achieved a high accuracy rate, proving the efficiency of transformers in rising cybersecurity text classification with minimal fine-tuning.

Hanxiang et al. [31] observed the application of LLMs in cybersecurity, emphasizing their application in threat detection, vulnerability assessment, and automated response by performing a systematic literature review. It reviewed findings from existing research, presenting models used like GPT and BERT, and demonstrated outcomes across various tasks. However, the paper identified critical challenges, including data quality, model interpretability, and adversarial robustness, which can be addressed in future research.

Ćirković et al. [32] investigated the utility of a fine-tuned GPT-2 model to generate synthetic payloads of web application penetration testing. The aim was to increase and automate the accuracy of detecting vulnerabilities such as SQL Injection, Cross-Site Scripting (XSS), and Command Injection. Utilizing a Flask-based web interface, the scheme permits penetration testers to choose attack sorts and accept tailored payloads at the same time. The fine-tuned GPT-2 model was trained on a domain dataset, and the evaluation result showed that the loss values reached between 1.42 and 1.58, representing high accuracy in generating true attack inputs. Overall, this paper provides a useful tool for cybersecurity testing, using LLM to detect threats more effectively.

A dataset with a total of 54,928 instruction-response pairs, created to test the safety and performance of LLMs in cybersecurity, was introduced by ElZemity et al. [33]. Seven open-source LLMs involving Gemma 2 9B and Llama 3.1 8B, using the dataset, evaluated their safety by utilizing the OWASP Top 10 framework. Findings presented an important decline in safety flexibility after fine-tuning. For instance, Llama 3.1 8B’s resilience to rapid-injection attacks dropped from 0.95 to 0.15. Regardless of this, the models established resilient task implementation on the CyberMetric benchmark, reaching up to 92.5% accuracy. Consequently, CyberLLMInstruct suggests a helpful benchmark for the secure and efficient deployment of LLMs in cybersecurity.

Levi et al. [34] presented a SecKnowledge model, which is an instruction-tuning dataset precisely proposed using domain knowledge to improve LLMs in cybersecurity. The study fine-tuned diverse LLMs, mentioned as CyberPal.AI, applying this dataset, and the performance evaluation was done with SecKnowledge-Eval, which is a comprehensive cybersecurity benchmark. The findings confirmed an improvement of up to 24% over standard models, representing the success of the expert-driven instruction dataset. This paper focuses significantly on enhancing AI-based cybersecurity.

This research shows the varied purposes of the synthetic dataset generation based on cybersecurity, ranging from phishing email generation and anomaly detection to cyber exercise content creation. It presents a foundation for the use of LLMs in enhancing cybersecurity processes. Table 3 summarizes Synthetic dataset generation research based on the approach used, IoC types, and results.

Although previous studies, such as [26,27,29,30,31], have investigated the utility of LLM in cybersecurity in different field such as synthetic network traffic, synthetic payload generation, instruction dataset design, and safety evaluation of fine-tuned models; this study displays different methods. However, our research utilized Gpt.3.5 in combination with real IoC domain knowledge. The fine-tuned GPT model was utilized to generate a synthetic dataset labelled by IoC type and social media platform name, which was curated and preprocessed from public social media comments datasets. In comparison to prior studies that relied on curated or expert-generated datasets, our approach used real-world user-generated content, increasing contextual relevance and variety. Additionally, it utilized both ML and DL models to evaluate the quality and efficacy of the generated data. This multidimensional approach contributes a novel approach by associating generative AI with threat intelligence sourced from open sources, presenting valued insights into the dynamic and user-driven nature of current threats.

## 3. Implementation and Results

**Figure 1:**
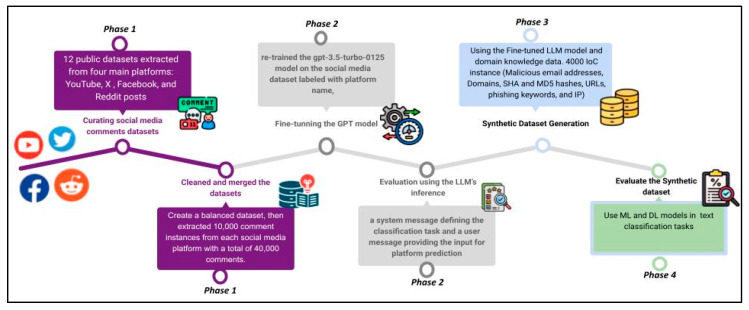
Proposed model pipeline.

This section presents the methodology and implementation pipeline, as well as the results. Generally, the methodology involves four phases: (1) curating real and diverse social media comments datasets, then cleaning and labelling them using the platform name, (2) fine-tuning and evaluating a version of ChatGPT 3.5 on the dataset to classify its instances by platform, (3) utilizing an IoC domain-knowledge and the previously fine-tuned GPT model to generate a synthetic dataset labelled for both social media platform and IoC type, (4) evaluating the performance of selected ML and DL models on the generated dataset to predict IoCs associated with social media comments. Figure 1 illustrates the proposed pipeline. 

### 3.1. Phase 1: Curating Social Media Comments Datasets and Labeling Them

Initially, the researchers curated 12 public datasets, which provide a comprehensive representation of social media communications. These datasets were extracted from four main platforms: YouTube, X, Facebook, and Reddit posts. The YouTube and X datasets are short in contrast to the ones extracted from Facebook and Reddit. Moreover, the datasets vary in size. For instance, the X datasets in total included over a million posts, while Reddit comments were 13,782. We ensured the collection of comments from various platforms with different topics to allow for variability of content being generated. Table 4 presents a detailed description of the curated datasets, their topics, and size.

The researchers cleaned the datasets, which included removing unneeded columns, and kept only the comment column, resulting in a merged dataset of 1,335,814 social media comments. To create a balanced dataset, the researchers extracted 10,000 comment instances from each social media platform, with a total of 40,000 comments. Table 5 and Table 6 provide a statistical description of this merged and balanced dataset, considering comment length and word count per platform.

The final version of the dataset has been automatically labelled using the platform name (X Facebook, YouTube, Reddit), and a column was generated for that. The objective is to assign each comment a style that defines its scope, potential content, and domain.

### 3.2. Phase 2: Fine-Tuning the LLM (GPT 3.5-Turbo) Model on the Merged Dataset

The main objective of LLM fine-tuning (in this case, GPT 3.5-Turbo) is to learn the natural style and tone of the social media comments from the original publicly curated dataset, then test the LLM model’s accuracy in classifying the comments based on the style of each platform. Learning the textual representation of the dataset is a crucial step later when generating the synthetic dataset.

To prepare the dataset for fine-tuning, it was first split into training (80%) and testing (20%) datasets using stratified sampling to ensure the representation of comments across all classes (platforms). Next, the dataset is structured into a conversational format following the representation required by OpenAI fine-tuning jobs, where each record transforms into a dictionary that captures the interactions between messages for each of the following components: system, user, and assistant. The role of the assistant is defined as classifying social media comments by platform. As for the user message, it is basically the comment input that needs to be classified, and the assistant message represents the targeted classification output.

**Figure 2:**
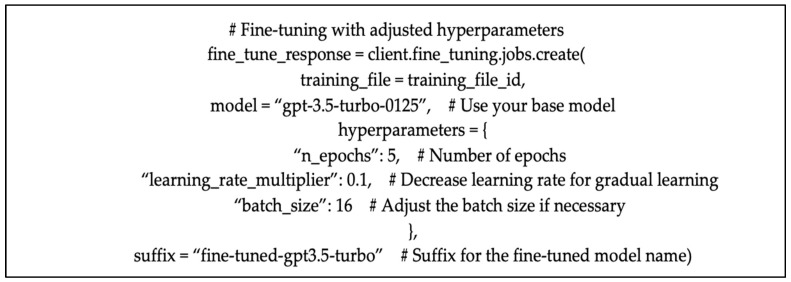
Hyperparameters of the fine-tuning job.

Considering recent advancements in LLMs, developers can now, for example, train the available OpenAI models on their own datasets to perform mainstream tasks like classification or synthetic data generation. This process entails using the “client.fine_tuning.jobs.create” function as a call to the language model using OpenAI’s API (Figure 2). This function creates a fine-tuning job by sending a properly formatted dataset to the API, along with configuration parameters specific to the fine-tuning task. Fine-tuning text generation models on the user-provided dataset helps obtain results with better quality compared to simple prompting, as well as train more examples than a direct text prompt can contain. So, basically, during this phase of the implementation, the researchers re-trained the GPT-3.5-turbo-0125 model on the social media dataset labeled with platform name, hence providing the model with a variety of realistic examples. The fine-tuning process saves the updated weights as a new model checkpoint [35,36]. At this stage, fine-tuning the LLM was intended to help it learn the style, tone, format, and various qualitative aspects that distinguish social media comments coming from different platforms.

For an actual fine-tuning task, the GPT-3.5 base model (gpt-3.5-turbo-0125) was used to learn the features of the dataset, with specific hyperparameters including setting the number of epochs to 5. A learning rate multiplier parameter is included as well and set to 0.1, which ensures gradual learning by reducing the learning rate in comparison to the base model. This leads to a smoother model convergence. Additionally, the batch size has also been set to 16, which impacts both speed and stability of training, resulting in better optimization and model generalization. The fine-tuning job in our implementation ran for 2 h and 18 min.

**Figure 3:**
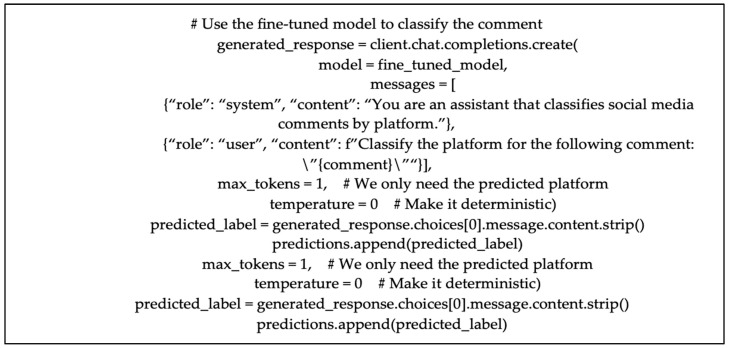
Using the fine-tuned model for classification.

Once fine-tuning is complete, the resulting model is evaluated on the test dataset, which contains unseen examples that the model has not encountered during training (Figure 3). This ensures that the evaluation reflects the model’s ability to generalize beyond the training data. As we are using an LLM for a classification task, the prompt must be phrased in a way that ensures the required output. The evaluation of the fine-tuned model is performed using the LLM’s inference capabilities, where a system message defines the assistant’s classification task and a user message provides the input (or the social media comment to classify). The assistant is supposed to output the predicted platform. The predictions are compared to the true platform labels to calculate performance metrics, including accuracy, precision, recall, and F1 score.

The classification task was completed within a total time of 33.36 min. A CPU usage of 16.4% and memory usage of 14.3% indicate a moderate use of computational resources, hence indicating an efficiently executed task without much straining of the system’s resources. The fine-tuned model’s performance was evaluated using the metrics of accuracy, precision, recall, and F1 score. The model achieved 91.05% accuracy, which indicates that the model was able to correctly classify the platform for the test set. Additionally, both a precision score of 91.32% and a recall value of 91.05% signify a robust detection capability. Similarly, an F1 score of 90.99% reflects a balanced performance between precision and recall, which further emphasizes the model’s ability to generalize well on unseen data.

### 3.3. Phase 3: Synthetic Dataset Generation Using the Fine-Tuned LLM and IoC Domain Knowledge

This phase consists of two steps: (1) developing an IoC domain knowledge dataset, which will be (2) used as an embedding to generate a synthetic dataset using the fine-tuned model (fine-tuned-gpt3-5-turbo:AoBGnG9C). The objective of this phase is to curate a dataset of IoCs that demonstrate various cybersecurity risks and use it to generate a synthetic social media comments dataset, which includes an IoC per comment. The resulting dataset is labeled for both platform and IoC type, in addition to the body of the comment that demonstrates the use of the IoC.

The domain knowledge includes both historical and updated indicators sourced from public forums, reports, and websites, providing a comprehensive and accurate representation of cybersecurity threats, including a total of 4000 instances of IoC (500 instances per IoC type). The collected indicators consist of email addresses associated with malicious or suspicious activities, malware domains linked to malware distribution and phishing, and hashes (SHA-1, SHA-256, and MD5) susceptible to collisions and attacks, malicious URLs and malicious IPv4 addresses used for network communication and geolocation of threats, and phishing keywords commonly used to attract victims. From these types, a comprehensive database was prepared to be used in the synthetic dataset generation. The dataset consisted of two columns, one for the “ioc_instance” and the other for its “type”.

The demand for domain knowledge to develop realistic cybersecurity threat datasets is important, as the scarcity of IoC-labeled datasets is a recognizable issue. The primary goal of using these indicator files is to enhance the LLM’s capability to generate synthetic datasets that accurately represent the multifaceted nature of real-world cyber threats. This methodology significantly contributes to the advancement of cybersecurity research by improving the detection, analysis, and mitigation of both existing and emerging threats. Table 7 explains each indicator, indicating its size and type while providing some examples.

The actual implementation of this step entails several platform-specific attributes that will drive the generation process; for example, the maximum number of tokens to be generated and some notes on the style and tone of each platform. The IoC dataset is then embedded, and a generate function is created using the OpenAI API to utilize the fine-tuned GPT by iterating over the specified platforms and IoCs, ensuring that no IoC type is repeated for a particular platform, to ensure diversity in the generated comments.

**Figure 4:**
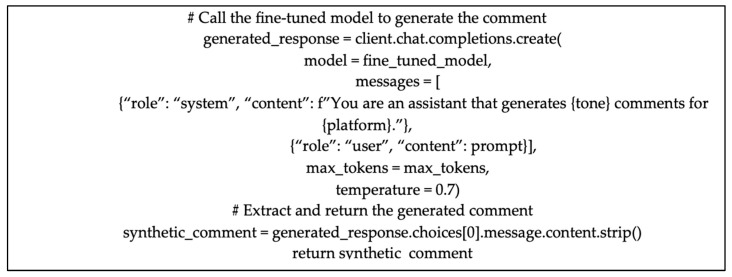
Prompt to generate synthetic dataset, including domain-knowledge IoCs.

For each IoC type, the function selects a corresponding IoC instance and builds a prompt that is sent to the LLM. The prompt is engineered in a way that provides the model with direct and clear instruction, as well as allows for dynamic generation of max_tokens as the per-defined maximum value for each platform. It is composed in such a way that it emphasizes the context-specific nuances required for each platform. For instance, a comment generated for X might be phrased as “Generate an X comment about [IoC]. The comment must be casual and include emojis,” while a similar prompt for Reddit would be more detailed and discussion-oriented in style. This prompting method ensures that the model generates contextually relevant comments that emulate the conventions of each social media platform. The temperature hyperparameter was set to 0.7 to allow for more creativity in the generation process (Figure 4).

We experimented with generating two datasets with different sizes, one including a total of 4000 instances (125 instance per IoC type/per platform), and another dataset that consisted of a total of 12,000 instances (375 per IoC/per platform). Table 8 and Table 9 provide a statistical description of the generated synthetic dataset, considering comment length and word count per platform.

### 3.4. Phase 4: Evaluating ML and DL Models on the Generated Dataset to Classify Comments by IoCs

In this last stage of the implementation, the researchers investigated how ML and DL models can classify the two generated synthetic datasets, and whether these models will accurately predict the IoC class of each comment.

Four models were selected for this implementation: Logistic Regression (LR), SVM, MLPClassifier, and DenseNN. These models have different approaches to text classification; LR, for example, is a linear model, which predicts categorical classes by providing weights to input features. To ensure convergence during its training on the TF-IDF-transformed text data, the LR’s initialization is set to “max_iter = 1000”. With regards to the SVM, it can identify the optimal hyperplane, which maximizes the margin between classes, using kernels that can handle non-linear relationships. In our implementation, it is trained on the TF-IDF features, which handle high-dimensional and sparse data in text classification, particularly when class boundaries cannot be identified.

To address the complexity of patterns in textual data, DL models like MLPClassifier and DenseNN are used. MLPClassifier, being a feedforward neural network, can learn non-linear patterns utilizing multiple hidden layers and backpropagation. In this study, this model was configured with a maximum of 500 iterations to effectively handle the intricate relations between TF-IDF features and output classes, yet with higher computational requirements. The DenseNN implementation, on the other hand, includes three dense layers with ReLU activation and a softmax output layer for multi-class classification. The Adam optimizer is used, and the model is trained on the TF-IDF data. This model is suitable for this type of dataset due to its hierarchical feature learning capability, which allows it to capture deeper and more abstract relationships in textual data.

These four models were trained and tested on two synthetic datasets, one with 4000 instances and the other with 12,000. Table 10 and Table 11 demonstrate the performance of four algorithms on the 4000-instance synthetic dataset using the accuracy, precision, recall, and F1 score metrics, whereas Table 12 and Table 13 demonstrate their performance on the 12,000-instance synthetic dataset.

The preprocessing of both datasets included transforming the text of the comments into numerical features using TF-IDF to capture word significance in the dataset, as well as reducing dimensionality. We performed a stratified train/test split (80%/20%) to ensure that both train and test include a balanced distribution of labels (by platform name).

Considering the evaluation results on the 4000-instance dataset, DenseNN had the highest accuracy (77.25%) overall, then LR (76.75%) and SVM (75.75%), respectively. DenseNN also exhibited the highest overall precision (77.88%) and F1 score (77.43%), while LR maintained a strong balance between them: (76.90%) and (76.75%). SVM performed well in comparison, with slightly lower precision (76.28%) and recall (75.75%) but higher computational efficiency. MLPClassifier, with the lowest (72.63%), demonstrated consistent overall metrics across IoC classes. Generally, the results of the DenseNN implementation indicated the best resource efficiency, with the shortest training time (3.99 s) and the lowest memory usage (8.5%).

Results of the classification by class indicate that DenseNN’s performance was better compared to other models, especially on such classes as sha1 (100% precision, 95% recall) and domain (98% precision, 96% recall). LR and SVM performed best on predicting sha256 (98% precision for both) and domain (98% precision). The models’ lowest performances were in the keyword and Ip classes. For example, DenseNN achieved the highest precision and recall for keyword (53% precision, 45% recall) and ip (44% precision, 51% recall) compared to LR and SVM, which struggled with these classes due to their limitations in capturing non-linear patterns. In this in-class analysis, DenseNN achieved the highest precision and recall values compared to other models.

When implementing the same pipeline on the 12,000-instance dataset, the overall accuracy of LR proved to be the highest (82%), which outperformed other models. Moreover, its consistently high precision (81.69%) and recall (81.83%) across all classes indicate robust generalizability. SVM followed closely, achieving an accuracy of 81.5%, just slightly below that of LR, as it can handle high-dimensional feature spaces. Nonetheless, SVM required more computational time compared to LR, indicating its inherent complexity.

As a DL model, the (DenseNN) achieved an accuracy of 80%, showcasing its ability to model complex, non-linear relations in the dataset, as it has a layered architecture that enables it to extract intricate patterns, providing accurate predictions even when these relations are not explicitly defined. Considering computational efficiency, DenseNN achieved this performance with a training duration of only 8.23 s compared to other DL models. The MLPClassifier was next with an accuracy of 79.54%. This model’s performance indicates its applicability to handling complex datasets, yet its significantly higher training duration (108.53 s) suggests its lesser suitability for real-time or large-scale applications.

Investigating the precision and recall of the in-class classification, we noted that amongst the ML models, LR demonstrates consistently high precision and recall, particularly high performing in domain prediction with a precision of 0.98 and recall of 0.93. SVM demonstrated comparable performance, achieving the highest precision (0.99) for SHA1 and domain classes, while showcasing a balanced recall across all categories. The MLPClassifier achieves perfect precision (1.00) for SHA1, showcasing its ability to capture complex patterns, but it slightly underperforms in the classification of IP and email classes compared to other models. The DL model, DenseNN, outputs a strong recall value for SHA1 (1.00) and maintains a balanced performance across other classes.

## 4. Conclusions

The study discussed the challenges inherent in traditional methodologies for classifying IoCs due to the scarcity of labeled datasets. These datasets are essential for understanding a wider range of threats that are present on many digital platforms, including social media. Therefore, the present study investigated the feasibility of using the fine-tuning features of OpenAI’s LLMs in combination with IoC-curated domain knowledge to generate semirealistic synthetic textual datasets, which incorporate IoCs as well as label these text instances using select IoC types.

The previously discussed results are crucial in any future discussion of CTI systems and applications, as the availability of pre-trained predictive models of IoCs in textual contexts can prove to be useful not only for the detection and extraction of social media-based threats but also in other types of organizational scenarios.

Future research in this domain can focus on key areas that might lead to the enhancement of the detection of IoCs in various contexts. Adversarial and reinforcement learning techniques can be utilized by researchers to learn both to generate synthetic datasets using LLMs and extract IoC features from text datasets. Moreover, one promising enhancement can be through the utilization of multi-agent LLMs in scenarios of generating synthetic datasets, as well as detecting IoCs. The integration of multi-modal data sources, such as combining textual information with metadata and network traffic data, can further enhance IoC detection accuracy. Future studies may also explore the use of graph-based learning techniques to capture complex relationships between IoCs across different platforms.

## Figures and Tables

**Table 1:** Summary of studies based on cybersecurity threat detection.

Ref.	Domain	Objective	Models	Results
[13]	DT in Cyber–Physical Systems	Review risk auditing frameworks for DTs	Threat Classification	Cyber attacks and data integrity are the main risks
[14]	IoT	Classify threats and countermeasures in IoT	Layer-wise threat classification and mitigation	DoS and phishing dominate; need for layered defenses
[15]	Workstation Environments	Detect threats and mitigation in workstation domains	Risk Analysis—Content Classification	Malware and MitM attacks are popular access control critical
[16]	ML in Cybersecurity	Review ML models for threat detection	Supervised ML, Unsupervised, and Anomaly Detection	SVMs, Decision Trees are effective; ML for detection
[17]	WSNs	Evaluate threats and lightweight protections for WSNs	Qualitative Analysis and Energy-efficient defenses	DoS, Sybil attacks are prominent and required for lightweight security

**Table 2:** Summary of studies based on IoCs.

Ref.	Model/Approach	Features Used	IoC Types	Data Sources	Evaluation Result
[18]	Neural-based sequence labeling (LSTM + CRF)	Spelling, Contextual Features	Emails, IPv4, Domain, Malware, URL, Vulnerability, File Hashes, File Info, Attacker, Attack Method, Attack Target	Report	90.40%
[19]	Neural network with multi-head self-attention	Contextual Features from Unstructured Text	Emails, IPv4, Domain, Malware, URL, Vulnerability, File Hashes, File Info, Attacker, Attack Method, Attack Target	Report	93.1% (English), 83% (Chinese)
[20]	Convolutional Neural Network (CNN)	For evaluation used correctness, timeliness, and overlap	IP addresses, URLs, file hashes, domain addresses, and CVE IDs	Twitter	URLs most frequently traded; 48.67% malicious.
[21]	CNN for Classification and BiLSTM for NER	Multi-task learning approach	Security-related information	Twitter	94%
[22]	combination of graph theory, ML, and text mining techniques		Malicious URL	Twitter	90% of URLs
[23]	SVM	Text-based keywords	URL	Dark web	Precision 83%, recall 90%, and F-measure 96%
[24]	SVM	Developed a framework using Wikitology	Vulnerabilities and attacks	Web text	evaluated against descriptions from the National Vulnerability Database 89.47%
[25]	Bagging Decision Tree: NB, KNN, DT, BDT, RF, ET, ABC, GB, VE	Shared ML Model Features	Five different simulated cyber-attacks: Man-in-the- Middle Change attack (MITM_C), Man-in-the-Middle Read attack (MITM_R), Modbus Query Flooding, Ping Flood DDoS, TCP SYN Flooding DDoS.	Industrial Control System (ICS) testbed,	BDT accuracy 94.24%, precision 95%, recall 93% and F1-score 94%
[26]	Topic extraction, semantic similarity computation and multi-label machine learning classification	latent semantic indexing method, semantic similarities	Threat Action	Threat-related articles.	precision59.50%, recall 69.86% and F1 56.96%.
[27]	Sharing ML models as IoCs	Network Traffic Data Contextual Information	Integrated with existing CTI platforms such as MISP, TheHive, and Cortex.	Network Traffic—CTI data	-

**Table 3:** Summary of studies based on synthetic dataset generation.

Ref.	Model/Approach	IoCs Type	Evaluation Result
[9]	Natural Language Generation (NLG)	Malicious emails	Among SVM, (NB), and (LR). (LR) achieved the highest accuracy at 91%.
[10]	Named entity recognition (NER) and topic extraction are used to structure the information based on a novel ontology called the Cyber Exercise Scenario Ontology (CESO)	Create new exercise scenarios	80% was achieved in the F1 Score
[28]	evaluated NLP-based anomaly detection systems	Creating synthetic cyber threats	Identify the benefits and limits of these methodologies
[29]	Used LLM-based packet generator and evaluated its performance using metrics like loss, accuracy, and success rate.	Synthetic network traffic	Transformers are effective for synthetic packet generation with minimal fine-tuning.
[30]	combined GPT and BERT models to enhance text classification in cybersecurity	Cybersecurity-related text	Not provided
[31]	Literature review of the application of LLMs	threat detection, vulnerability assessment	Not provided
[32]	Fine-tuned GPT-2 for payload generation	Synthetic payloads: XSS, SQL, Command Injection	Evaluation loss: 1.42‚ Äì1.58; successful generation of realistic payloads
[33]	Fine-tuned multiple LLMs (Llama 3.1 8B, Gemma 2 9B, etc.) using CyberLLMInstruct	Safety analysis: Prompt injection, data exposure, unauthorized actions	Llama 3.1 8B‚ and safety score dropped from 0.95 to 0.15. performance improved to 92.5% on CyberMetric
[34]	Expert-driven instruction dataset (SecKnowledge) for fine-tuning LLMs (CyberPal.AI)	Instruction-following tasks for incident response, threat hunting	Performance improved up to 24% over standard models

**Table 4:** Curated social media dataset information.

Platform	Sources	Datasets	Columns Count	Size	Total
YouTube	Hugging face	Comment on YouTube video	1	180,000	180,000
X	Kaggle(K) Data.world (D)	2. Customer Support (K) 3. Apple twitter sentiment (D) 4. Customised data (API) named combined1	14 12 1	1,048,576 3886 10,752	1,063,214
Reddit	Kaggle	5. Recipes (918)(862)(671) = (2450) 6. Depression Cleaned 7. Conspiracy Theories 8. Explore Reddit’s Funny Subreddit 9. Reddit’s 2400 Posts	18 12 8 8 10	2450 7650 1198 957 1527	13,782
Facebook	Kaggle	10. Political Advertisements 11. Transport for NSW 12. Spam or Ham—EMP Week 2 ML HW	24 12 3	72,787 458 5573	78,818

**Table 5:** Descriptive statistics of comment length in the merged dataset.

Platform	Mean	Std	Min	Median	Max
Facebook	85.5572	74.210551	2	63	1868
Reddit	976.3268	1815.243355	1	373	32,575
X	115.3024	55.121442	5	115	372
YouTube	163.2189	329.854037	1	76	9017

**Table 6:** Descriptive statistics of word count per platform.

Platform	Mean	Std	Min	Median	Max
Facebook	16.4207	13.21138	1	13	273
Reddit	170.7618	294.032278	1	70	5632
X	19.5842	10.041852	1	19	66
YouTube	27.8229	56.015892	1	13	1760

**Table 7:** IoC dataset description.

IoCs	Description	Size	Type	Example
Malicious email addresses	It contains a public email associated with malicious or suspicious activity	500	string	4dminone@gmail.com 56465sdfsd@kksdfs.com 567f27df@opayq.com
Domains	It contains domains, which are associated with malware, phishing, or other malicious activity which can be used in a variety of cyber attacks, including virus distribution, phishing website hosting…etc.	500	Text string	0.0.0.winrar.fileplanet.com bestdove.in.ua sus.nieuwmoer.info
SHA-1, SHA-256, and MD5 hashes	They are susceptible to collision and attacks, which may compromise data integrity, allow password cracking, and enable digital signature forging, posing serious security issues.	500 per hash type	hexadecimal strings	SHA-1 “18b0e41e2835c92572d5 99e6e4e0db63e396cf7f” SHA-256 “83d1e736a793ba5d 14b51f8ef8310bf13a2591fc40ad 34d1fa3e74a cf5d40c70” MD5 “fed87b42561dafe8a9ee8c5ffa2e434e”
URLs	It includes domains and subdomains, some of which appear suspicious and could be linked to phishing, malware distribution, or other harmful activity.	500	Text strings	http://gbdgs.blogspot.bg/ http://fwgnu.blogspot.fi/ http://fwsbf.blogspot.hr/
IP addresses	It consists of IPv4 addresses, which are required for network communication, device identification, and geolocation.	500	dotted-decimal	190.89.103.40 190.173.76.130 190.216.227.18
Phishing keywords	It consists of terms and phrases commonly associated with phishing attempts to lure victims into taking actions that compromise their security	500	text	Reserves the right for only information you requested

**Table 8:** Statistical description of 4000 instances.

Platform	Count	Mean	Std	Min	25%	50%	75%	Max
Facebook	1000	70.059	27.64156	13	51	67	86	212
Reddit	1000	637.689	126.8704	216	554.25	664.5	733	912
X	1000	65.207	22.07336	10	50.75	65	79	150
YouTube	1000	223.657	50.84705	88	190	220	254	455

**Table 9:** Statistical description of 12,000 instances.

Platform	Count	Mean	Std	Min	25%	50%	75%	Max
Facebook	3000	70.082	27.23251	13	51	67	86	212
Reddit	3000	641.0153	125.6913	61	562	671	734.25	921
X	3000	64.61267	21.34391	10	51	64	78	150
YouTube	3000	223.897	52.68712	84	189	220	254	492

**Table 10:** The performance result of four algorithms on the 4000-instance synthetic dataset.

Model	Training Duration (s)	CPU Usage (%)	Memory Usage (%)	Accuracy	Precision	Recall	F1 Score
ML models							
Logistic Regression	5.11	99.9	10.9	0.7675	0.769	0.7675	0.7667
SVM	4.17	15.3	10.9	0.7575	0.7628	0.7575	0.7581
DL models							
MLPClassifier	55.76	99.9	10.7	0.72625	0.735	0.72625	0.7298
DenseNN	3.99	22.2	8.5	0.7725	0.7788	0.7725	0.7743

**Table 11:** In-class Results of Synthetic Dataset Classification by IoC.

Model	Metric	URL	sha256	sha1	md5	Keyword	Ip	Email	Domain
Logistic Regression	Precision	0.85	0.98	0.95	0.83	0.5	0.48	0.58	0.98
	Recall	0.93	0.85	0.93	0.91	0.52	0.41	0.64	0.95
SVC	Precision	0.83	0.98	0.99	0.78	0.5	0.45	0.59	0.98
	Recall	0.91	0.84	0.91	0.9	0.53	0.4	0.64	0.93
MLPClassifier	Precision	0.84	0.9	0.98	0.87	0.45	0.41	0.47	0.97
	Recall	0.9	0.86	0.95	0.79	0.43	0.43	0.53	0.92
DenseNN	Precision	0.87	0.98	1	0.88	0.53	0.44	0.55	0.98
	Recall	0.95	0.89	0.95	0.9	0.45	0.51	0.57	0.96

**Table 12:** Overall Accuracy Results of Synthetic Dataset classification by IoC (size = 12,000).

Model	Training Duration (s)	CPU Usage (%)	Memory Usage (%)	Accuracy	Precision	Recall	F1 Score
ML models							
Logistic Regression	10.86	99.9	5.1	0.8183	0.8169	0.8183	0.8167
SVM	26.68	15	5.2	0.8158	0.8164	0.8158	0.8146
DL models							
MLPClassifier	108.53	99.9	5.2	0.7954	0.8008	0.7954	0.7978
DenseNN	8.23	22.1	6.3	0.8029	0.8064	0.8029	0.8044

**Table 13:** In-class Results of Synthetic Dataset Classification by IoC (size = 12,000).

Model	Metric	URL	sha256	sha1	md5	Keyword	Ip	Email	Domain
Logistic Regression	Precision	0.92	0.97	0.98	0.84	0.6	0.58	0.66	0.98
	Recall	0.93	0.96	0.99	0.91	0.63	0.49	0.72	0.93
SVM	Precision	0.91	0.98	0.99	0.84	0.58	0.58	0.66	0.99
	Recall	0.94	0.94	0.98	0.91	0.65	0.47	0.71	0.92
MLPClassifier	Precision	0.91	0.96	1	0.9	0.53	0.52	0.61	0.98
	Recall	0.92	0.96	0.97	0.86	0.58	0.5	0.65	0.93
DenseNN	Precision	0.9	0.96	0.99	0.91	0.57	0.5	0.63	0.99
	Recall	0.93	0.96	1	0.87	0.58	0.51	0.65	0.92

## Data Availability

The raw data supporting the conclusions of this article will be made available by the authors on request.
